# Evaluation of Specific *Torulaspora delbrueckii* Fractions to Stimulate Malolactic Fermentation in Limiting Conditions

**DOI:** 10.3390/microorganisms13102391

**Published:** 2025-10-17

**Authors:** Aitor Balmaseda, Paloma Toraño, Benjamin Leroux, José María Heras, Sibylle Krieger-Weber, Nicolas Rozès, Magali Deleris-Bou, Cristina Reguant

**Affiliations:** 1 Grup de Biotecnologia Enològica, Departament de Bioquímica i Biotecnologia, Facultat d’Enologia, Universitat Rovira i Virgili, C/Marcel·lí Domingo s/n, 43007 Tarragona, Spain; paloma.torano@estudiants.urv.cat (P.T.); cristina.reguant@urv.cat (C.R.); 2 Lallemand SAS, 19 Rue des Briquetiers, 31700 Blagnac, France; bleroux@lallemand.com (B.L.); jmheras@lallemand.com (J.M.H.); skrieger@lallemand.com (S.K.-W.); mbou@lallemand.com (M.D.-B.); 3 Grup de Biotecnologia Microbiana dels Aliments, Departament de Bioquímica i Biotecnologia, Facultat d’Enologia, Universitat Rovira i Virgili, C/Marcel·lí Domingo s/n, 43007 Tarragona, Spain; nicolasrozes@urv.cat

**Keywords:** malolactic fermentation, lactic acid bacteria, *Oenococcus oeni*, non-*Saccharomyces*, *Torulaspora delbrueckii*, yeast fractions, mannoproteins

## Abstract

Malolactic fermentation (MLF) is a bioprocess driven by lactic acid bacteria (LAB), which is desired in red and highly acidic white wines. Among all LAB, *Oenococcus oeni* is the main species in wine, followed by *Lactiplantibacillus plantarum*. The harsh conditions found in wine—not only due to the low nutrient concentration but also the presence of antimicrobial compounds such as ethanol, high acidity, SO_2,_ and polyphenols—can compromise MLF performance. In recent years, the use of certain non-*Saccharomyces* yeasts, such as *Torulaspora delbrueckii* or *Metschnikowia pulcherrima*, as starter cultures for alcoholic fermentation, has emerged as a promising strategy to improve MLF. In this study, we evaluated the effect of four different fractions from a *T. delbrueckii* strain on MLF performance. First, the positive impact of this strain as a starter culture on *O. oeni* growth was confirmed; then, yeast-derived compounds were tested in different wines. Two fractions showed the most promising results in reducing MLF duration: the inactivated yeast fraction and the autolysate fraction. Those enhanced bacterial viability and promoted mannoprotein consumption. These findings highlight the potential of *T. delbrueckii*-derived compounds as enological tools to support MLF under restrictive wine conditions.

## 1. Introduction

Malolactic fermentation (MLF) is a biotransformation driven by lactic acid bacteria (LAB) in wine. It consists of the decarboxylation of L-malic acid into L-lactic acid [1,2] that generally occurs after alcoholic fermentation (AF). As MLF reduces acidity, it is highly recommended in red winemaking and also for high-acidity white wines. Additionally, microbial stability is increased, and the organoleptic profile is modified [3]. During AF, wine yeasts consume nutrients and produce toxic metabolites, such as ethanol and medium-chain fatty acids; therefore, they leave a harsh environment for microbial growth. Among the enological yeasts, *Saccharomyces cerevisiae* is the dominant species from the middle to final fermentative stages [4]. It has been domesticated to the wine fermentative process, and it is part of the resident microbiota of cellars [5]. Nevertheless, during the first fermentative stages, a vast diversity of other yeasts, known as non-*Saccharomyces*, are predominant, which come from the grape skins and are subsequently found in grape must [6].

Also, some LAB have been progressively domesticated to this enological niche, being the most important the species *Oenococcus oeni* [7,8,9]. This bacterium has developed specific mechanisms to resist the wine environment, characterized by a low pH and moderate to high ethanol concentration, and to enable bacterial growth and survival in a very poor nutrient medium [10]. In this sense, L-malic acid degradation is one of those specific mechanisms for survival in wine found in LAB, which enables energy acquisition from L-malic acid decarboxylation.

After the completion of AF, wine yeasts undergo an autolytic process [11,12], in which they release some compounds to the wine, such as nitrogen compounds (amino acids, peptides, and proteins), vitamins, or mannoproteins, among others [13,14,15]. Thus, enriching wine with nutrients that can eventually be used by LAB. That is why, generally, MLF occurs after AF, when wine is enriched with those yeast-derived compounds. At this stage, yeast cells are no longer metabolically active, which reduces competition, and together with the release of yeast-derived compounds, creates favorable conditions for LAB growth and the initiation of MLF.

The harsh conditions found in wine can be somewhat counteracted by inoculating non-*Saccharomyces* for AF [16]. Their initial low population in grape must and their low ethanol resistance, together with the usual inoculation of *S. cerevisiae* in must, make it difficult to observe any changes in wine composition. In addition, as it is carried out for *S. cerevisiae*, these yeasts can also be inoculated at the beginning of AF to intentionally modulate wine characteristics [17,18,19,20,21,22,23]. Among the non-*Saccharomyces* yeasts, some strains of *Torulaspora delbrueckii*, *Metschnikowia pulcherrima*, and *Lachancea thermotolerans* are already available as commercial starter cultures [20]. These yeasts not only modulate wine organoleptic profile but also modulate other compounds that directly impact *O. oeni*, and consequently, impact the MLF performance [16]. Indeed, non-*Saccharomyces* can be inoculated as immobilized cultures [24,25], or even LAB that can help to counteract and protect the cultures from the harsh environment [26], or even yeasts and LAB together [27]. There are several authors who reported positive effects on MLF duration reduction using non-*Saccharomyces* in AF from different winemaking, mainly with strains belonging to *T. delbrueckii* [28,29,30] and *M. pulcherrima* [31,32] species. To this regard, some positive outcomes from the chemical modulation of wine composition by these two species are as follows: (i) the decrease in medium chain fatty acids, (ii) the increase in the pH value, (iii) the reduction in the ethanol content, (iv) the enhancement of mannoprotein release, and (v) the decrease in sulfur dioxide concentration, among other [33,34,35].

Interestingly, fractions from different enological yeast species present specific modulation on MLF duration. For instance, Balmaseda et al. [36] reported a general enhanced MLF when performing with yeasts’ lees of *T. delbrueckii*, but slowed down when fermenting in the presence of some *S. cerevisiae* yeast lees. In addition, the general outcome when supplementing wines with lees or yeast fractions is a positive reduction in MLF duration. In this sense, to our knowledge, there are no commercial non-*Saccharomyces* fractions for this purpose; they are all derived from *S. cerevisiae*.

Altogether, the aim of this work was to evaluate the potential use of some fractions obtained from a specific *T. delbrueckii* strain by different treatments on the MLF performance of *O. oeni* in some selected harsh red and white wines, characterized by low pH, high ethanol content, and high polyphenolic composition.

## 2. Materials and Methods

### 2.1. Wines, Yeast Fractions, and Microbial Strains

First, during vintage 2023, about 120 kg of Tempranillo grapes from the experimental cellar of the *Universitat Rovira i Virgili* were destemmed, and 60 kg were transferred each to two 100 kg steel tanks. Grape must general parameters were density 1091.8 g/L, primary amino nitrogen 57 mgN/L, ammonia 34 mgN/L, and pH 3.44. Sulfur dioxide in the form of K_2_S_2_O_5_ (Fisher Scientific, Madrid, Spain) was added to each tank at a final concentration of 20 g/hL. After one hour, tanks were inoculated: one with *S. cerevisiae* LMD38 (Lallemand SAS, Montreal, QC, Canada), and the other with *T. delbrueckii* LMD84 (Lallemand SAS, Montreal, QC, Canada), following rehydration instructions. Fermenting must was manually punched down every day, and samples were taken to monitor AF progression. After 48 h, NUTRIENT VIT^TM^ (Lallemand SAS, Montreal, QC, Canada) was added (20 g/hL). Then, *S. cerevisiae* was inoculated into the tank previously inoculated with *T. delbrueckii*. Once AF was finished (<2 g/L of residual sugars), wines were settled for 5 days at 4 °C.

Second, four wines from different cultivars were used in this study. These wines were provided by Lallemand SAS during vintage 2024, obtained after AF not heat-treated but stored at 4 °C before the experiment, representing harsh wines where MLF was performed. There were three red wines, including two Merlot wines (wine A and wine B) and a Tempranillo wine (wine C), and a single white Chardonnay wine (wine D). The initial composition of these wines is summarized in Table 1.

The fractions used in this study were also provided by Lallemand SAS. They were obtained with different treatments applied to the biomass of a specific strain of *T. delbrueckii* LMD84. The fractions were named as: inactivated yeast extract (Td_i_) resulting of a heat-treated cream (yeast cream at 70 °C for 15 min) then dried, autolysate extract (A) obtained after 20 h keeping the biomass at 55 °C followed by drying, yeast extract (YE), and cell wall extract (CW) were respective soluble and insoluble fractions resulting from the autolysis followed by a separation by centrifugation and then dried.

To perform MLF, commercial freeze-dried *O. oeni* strains (Lallemand SAS) were used following the manufacturer’s instructions, inoculating 1 g/hL of the product into each wine.

For the vinification of vintage 2023, LAA1, LAB6, LAB9, LAB2013, LAC20, and LAA4 *O. oeni* strains (Lallemand SAS) were used. For the subsequent trials, *O. oeni* LAB6 was used; then, other strains were used to confirm the observed effects in wine D: LAA1, LAB2013, LAA4, LAC20, and LAB9.

### 2.2. Experimental Fermentations

#### 2.2.1. Effect of *T. delbrueckii* as Starter Culture

*S. cerevisiae* control and *T. delbrueckii* racked wines were aliquoted in 24 different 50 mL-tubes and inoculated with the six *O. oeni* strains (1 g/hL), following the manufacturer’s instructions, in triplicate. Three tubes were left without inoculation. Approximately twice a week, samples were taken, and L-malic acid concentration was quantified by enzymatic methods (Analyzer Y15, Biosystems, Barcelona, Spain). When [L-malic acid] < 0.10 g/L, MLF was considered as finished, and the main oenological parameters were determined. Bacterial viability was monitored periodically on MRSmf plates (55 g/L MRS broth (BD™ Difco™, Fisher Scientific, Madrid, Spain), 4 g/L DL-malic acid, 5 g/L fructose, 100 mg/L nystatin and 20 g/L of agar ((Panreac Química, Castellar del Valles, Spain), pH 5.00).

#### 2.2.2. Screening of Different *T. delbrueckii* Fractions in Harsh Wines

Five aliquots of 50 mL of each wine (wines A, B, C, and D) were prepared and supplemented with 20 g/hL of each fraction (Td, A, YE, and CW). The remaining aliquot without supplementation was used as a control. After 24 h, 10 mL of wine were transferred to a 10 mL syringe (Luer-Lok™, BD, Madrid, Spain) coupled to a needle (0.8 × 40 mm, B. Braun, Melsungen, Germany) as described in Balmaseda et al. (2024) [37]. This syringe was used as a control for spontaneous fermentation. Then, the remaining volume was inoculated with 1 g/hL of *O. oeni* LAB6. Once inoculated, two additional syringes were filled with the inoculated wines, and each fermentation was performed in duplicate. The fermentations were incubated statically at 20 °C. Approximately twice a week, samples were taken, and L-malic acid concentration was quantified by enzymatic methods (Analyzer Y15, Biosystems). When [L-malic acid] < 0.10 g/L, MLF was considered as finished, and some oenological parameters were determined. After 65 days of inoculation, the unfinished MLF wines were considered stuck.

#### 2.2.3. Confirmation of Results in Harsh Wines

From the four studied wines in Section 2.2.1, wine C and wine D were selected to further characterize the effect of the fractions. The fractions Td_i_ and A were also chosen for this second trial, supporting the shortest MLF durations. Fermentations were carried out similarly to the previous section, inoculating LAB6 into wines C and D, supplemented with extracts Td_i_ and A. In this case, bacterial viability was determined before inoculation (t_0_), two, seven, and 14 days after inoculation by plating on MRSmf plates.

Similarly, the observed effects were confirmed in tubes containing 10 mL of wine D inoculated with LAA1, LAB2013, LAA4, LAC20, and LAB9. Wine D was supplemented with 20 g/hL of Td_i_ fraction. A control wine D without supplementation was also inoculated. Samples were taken weekly to measure L-malic acid concentration and bacterial viability. MLF was considered finished when <0.1 g/L of L-malic acid was determined. After 70 days, wines with the MLF not completed were considered as stuck fermentations. Fermentations were performed in duplicate.

### 2.3. Wine Characterization

pH was determined using a Crison micro pH 2002 pH-meter (Barcelona, Spain). D-lactic acid, L-lactic acid, acetic acid, citric acid, glycerol, ammonium, and PAN concentrations were determined with the analyzer Y15 (Biosystems, Barcelona, Spain).

Mannoprotein concentration was estimated by enzymatic measurement of mannose coming from a hydrolysis of the total precipitated polysaccharides with ethanol, following the method of Balmaseda et al. [38]. Briefly, 5 mL of wine was mixed with 5 volumes of 95% (*v*/*v*) ethanol and precipitated overnight at 4 °C. After washing the pellet twice with 10 mL of 95% ethanol, pellets were dried at 30 °C for 30′ in vacuum. Then, the pellet was resuspended in 1 mL of 5 M H_2_SO_4_, incubated at 90 °C for 1 h, and neutralized with 1 mL of 10 M NaOH. Finally, the sample was centrifuged (8500 rpm, 5′) and the supernatant was used to determine the free sugar concentration (D-glucose, D-fructose, and D-mannose) using the K-MANGL kit (Megazyme, Wicklow, Ireland). Concentration is expressed as mg mannose equivalents/L [35]. 

Amino acid quantification was performed as previously described in Balmaseda et al. [36]. In short, filtered samples were derivatized in borate buffer and methanol with the addition of L-2-aminoadipic acid as an internal standard, and incubated at 80 °C. The HPLC (Agilent 1100, Agilent Technologies, Waldbronn, Germany) was equipped with a DAD ultraviolet (Agilent Technologies, Waldbronn, Germany), and separation was performed on a Hypersil ODS C18 column (Agilent Technologies, Waldbronn, Germany) with a particle size of 5 μm (250 mm × 4.6 mm) at 20 °C, with sodium acetate buffer (pH 5.80) and an acetonitrile/methanol mixture as mobile phases. Amino acid concentrations were calculated against external calibration curves and normalized to the internal standard.

### 2.4. α-Mannosidase Activity

The determination of α-mannosidase activity was based on a protocol to measure β-glucosidase activity in *O. oeni* [39] with the modifications proposed by Toraño et al. (2024) [40]. Briefly, the mannosidase activity of all *O. oeni* strains was characterized in a wine-like medium. Two-milliliter samples were centrifuged, and the pellet was retained (corresponding to about 2 × 10^7^ CFU/mL) for analysis. The pellets were resuspended in 1 mL of 0.1 M sodium acetate buffer, pH 5.10. 25 μL of the resuspended pellet was added to 75 μL of substrate (25 mM p-nitrophenyl-α-D-mannopyranoside [p-NMan, (Sigma-Aldrich, Barcelona, Spain)] diluted in 0.1 M sodium acetate buffer, pH 5.10) and incubated for 30 min at 37 °C. Next, 100 μL of 1 M Na_2_CO_3_ was added. The reaction tubes were centrifuged, and the assay was read against the blank at 400 nm using a Spectro Star Nano (BMG Labtech, Ortenberg, Germany). From this measurement, the concentration of liberated p-nitrophenol (p-NP) was determined from a calibration curve taken from standard p-NP, where one unit of α-mannosidase activity (U) corresponded to 1 μmol of p-nitrophenol released per minute. The enzymatic activity (U) data were normalized to 1 L of culture. All the samples were collected to obtain the same population of 2 × 10^7^ CFU/mL, considering the growth curve (OD 600 nm vs. CFU/mL) of each strain. The viability of the samples was confirmed via CFU counting on MRSmf plates.

### 2.5. Statistical Analysis

All the statistical analyses of the results were performed using the statistics software XLSTAT version 2020.2.3 (Addinsoft, Paris, France). The analysis of variance was carried out by ANOVA with a subsequent Tukey HSD test to determine the significant differences between the samples: the confidence interval used was 95% and the statistical level of significance was set at *p* ≤ 0.05. The considered ANOVA factors were the supplementation with fractions of wine after MLF and the inoculated *O. oeni* strain; variables were the individual analytical results obtained in the experiment.

## 3. Results

### 3.1. Validating T. delbrueckii as a Malolactic Fermentation Enhancing Starter Culture

The Tempranillo wines produced during the vintage 2023 with *S. cerevisiae* or sequential fermentation with *T. delbrueckii* and *S. cerevisiae* finished after 10 and 16 days, respectively (Table 2). The AF duration is usually extended when using sequential or coinoculated AF strategies with non-*Saccharomyces* and *S. cerevisiae* strains [29]. Also, the potential reduction in MLF when inoculating *T. delbrueckii* was observed. In all inoculated wines, no matter the inoculated *O. oeni* strain, MLF was completed before it finished in the control wine, inoculated with *S. cerevisiae* only (Table 2).

The sequentially inoculated wine presented significantly lower ethanol content of about 13.6% (*v*/*v*) with respect to the control *S. cerevisiae* with about 14.1% (*v*/*v*). Slightly higher pH was measured in the sequentially inoculated wine (3.45 on average), compared to the control wine (3.41 on average), as observed by other authors [41]. Although these parameters are not definitive indicators of the potential benefits of *T. delbrueckii* on MLF, some of them have been reported to be closely associated with a positive effect on *O. oeni* [33].

The obtained wines were then inoculated with six different *O. oeni* strains. The MLF duration in the *T. delbrueckii* wines was significantly reduced by half in all *O. oeni* inoculated wines (Table 2). The observed results are related to the enhanced bacterial population found at the end of AF in those wines: about 10^5^ CFU/mL with respect to the 10^2^ CFU/mL of the control wine. This has been observed by several other authors when inoculating *T. delbrueckii* as a starter [42,43]. Under these conditions, the inoculation of commercial *O. oeni* strains did not notably accelerate MLF, possibly because the wines already harbored a substantial indigenous LAB population (around 7 × 10^5^ CFU/mL) prior to inoculation. Thus, it is possible that MLF in the *T. delbrueckii* wines was driven mainly by the native microbiota, although the inoculated *O. oeni* strains may also have contributed.

On the other hand, the bacterial population in the *S. cerevisiae* control wine was <10^2^ CFU/mL. In this wine, there was a significant reduction in the MLF duration when inoculating the six *O. oeni* strains.

The conditions found for this vinification were not as harsh as those of other wines with high acidity, high ethanol content, or high polyphenolic content [30]. The modulation caused by inoculating *T. delbrueckii* that enhanced *O. oeni* growth up to concentrations able to perform spontaneous MLF in a short time was confirmed. Based on these observations, the study proceeded to assess the potential benefits of supplementation with extracts derived from the same *T. delbrueckii* strain under more harsh conditions for *O. oeni* development, using wines produced exclusively with *S. cerevisiae* as the sole starter culture.

### 3.2. Screening of Different T. delbrueckii Fractions in Harsh Wines

In this second trial, we explored the effect of all four fractions from *T. delbrueckii* in four wines (Table 1), representing different matrices to perform MLF in harsh conditions (low pH, high polyphenolic index, or the combination of them, even low L-malic acid concentration). The initial bacterial population was <10 CFU/mL in wines B, C, and D. The population in wine A was about 5 × 10^3^ CFU/mL before inoculation.

**Figure 1:**
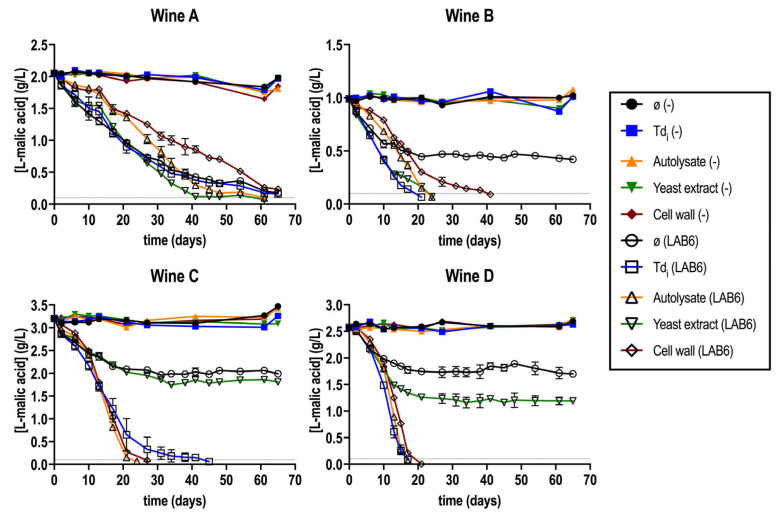
Malolactic fermentation (MLF) performance in wines (A–D) supplemented or not (ø) with all the *T. delbrueckii* fractions (Td_i_, A, YE, CW), inoculated with *O. oeni* (LAB6) or with no inoculation (−). The gray horizontal line represents [L-malic acid] = 0.1 g/L, considered as finished MLF. Fermentations were carried out in duplicate.

What was first observed was that wines supplemented or not with the different fractions were not able to start L-malic consumption without the inoculation of selected *O. oeni* strains (Figure 1). This confirms the harsh MLF scenario, in which a spontaneous MLF was not possible. In the inoculated wines, *O. oeni* started the L-malic acid consumption in all four tested wines, regardless of the supplementation. However, in all wines but wine A, the control fermentation with no fraction was stuck (Figure 1).

In general, there was a positive effect on the reduction in MLF duration in all wines when supplementing with the fractions (Figure 1) [40]. The only exception was wine A, where all the conditions behaved like the control with no addition. In this regard, wine A exhibited the lowest ethanol content and lower color intensity compared to the other two red wines (Table 1). Supplementation with the YE fraction resulted in stuck MLF in wines C and D, similarly to the control. In the case of the CW fraction, it prolonged MLF duration specifically in wine D compared to the control. This suggests that, although yeast-derived fractions can release compounds that potentially benefit MLF, not all fractions are equally effective in enhancing bacterial performance. In particular, the YE fraction exhibited a lower stimulatory effect on MLF progression compared to the other tested fractions. This observation is consistent with previous reports indicating that some yeast lees can, in certain cases, extend the duration of MLF beyond that of the control [36].

The general chemical characterization of the obtained wines after MLF was similar in all cases, except for the L-malic and L-lactic acid concentrations that depended on the completion of MLF (Table A1). As expected, wines in which MLF was completed exhibited higher pH values, regardless of fraction addition. This trend was also reflected in L-malic and L-lactic acid concentrations, which are directly related to MLF progression and, consequently, influence the pH [2]. Higher D-lactic acid concentrations were also observed in wines where MLF had been completed, independently of fraction supplementation. In addition, in two wines in particular, wine A and wine D, where MLF finished, showed lower citric acid concentrations and higher acetic acid levels. This is likely associated with increased metabolic activity of *O. oeni*, which commonly consumes citric acid during fermentation [1]. No clear trends were observed for glycerol. Regarding PAN and ammonium, interpretation is challenging because *O. oeni* can release these compounds through its protease and peptidase activity [44,45]. Nevertheless, wines supplemented with fractions tended to show higher levels of these compounds, independently of whether MLF was completed or not.

From all four wines tested, two were selected for further characterization: wine C and D, representing a harsh red (the one with the highest ethanol content) and a white wine, respectively. Fractions Td_i_ and A were also selected for the next experimentation since they showed the best MLF duration reduction (Figure 1).

### 3.3. Confirmation of the Potential of T. delbrueckii Fractions

**Figure 2:**
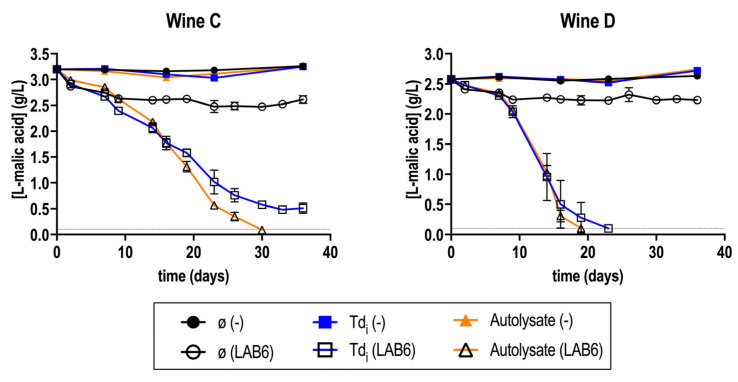
Malolactic fermentation (MLF) performance in wine C and D supplemented or not (ø) with *T. delbrueckii* fractions (Td_i_, and A), inoculated with *O. oeni* (LAB6) or with no inoculation (−). The gray horizontal line represents [L-malic acid] = 0.1 g/L, considered as finished MLF. Fermentations were carried out in duplicate.

Fermentations in wine C and D, supplemented with fractions Td_i_ and A, were repeated similarly to the previous section. In this case, fermentations were maintained for up to 40 days, obtaining similar results (Figure 2).

MLF in these wines was again difficult to finish. Spontaneous MLF did not occur, and fractions were necessary for MLF completion (Figure 2). This time, the supplementation with Td_i_ in wine C was stopped at [L-malic acid] = 0.5 g/L.

For a better understanding of the observed MLF dynamics, viability was determined after inoculation (t_0_), and after two, seven, and 14 days (Table 3). The initial viability was about 6 log CFU/mL in all wines and treatments. Then, *O. oeni* population increased during the first week, mainly in wine C (Table 3), with no statistical differences between the supplemented or non-supplemented wines. The differences came after two weeks of fermentation, when the wines supplemented with Td_i_ and A fractions showed an increased population of about 1 log compared to the control (Table 3). Hence, the addition of these two fractions improved the survival of *O. oeni* in wine conditions. This effect has also been observed when preparing *O. oeni* cells in the presence of mannoproteins prior to inoculation in synthetic wine [46].

Mannoproteins are glycoproteins present in the yeast cell walls that are released during AF and subsequent yeast autolysis [47]. They consist of 80–90% mannose polysaccharides and 5–10% protein [48].

The presence of mannoproteins has been associated with enhanced *O. oeni* survival, and this is related to late MLF stages [38,46,49,50]. These macromolecules constitute one of the potential key compounds in wine lees [47,51] for *O. oeni* [16]. The amino acid fraction or even the glycolytic one seems to have a positive impact on *O. oeni*. In this sense, it has been demonstrated that *O. oeni* has specific glycoside and peptidase activities that enable the release of sugars and amino acids [44,52,53], which could be used by the bacterium [52,54]. On the other hand, other authors have shown that mannoproteins could adsorb medium-chain fatty acids, thus detoxifying wine for *O. oeni* [55,56].

**Figure 3:**
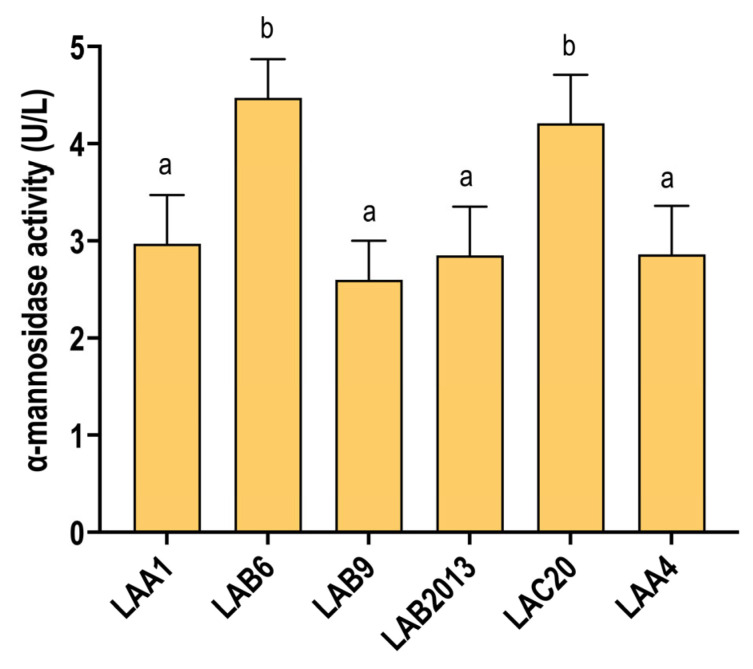
Mannosidase activity in the middle of MLF of the different *O. oeni* strains. Data are the mean values of triplicate assays expressed in units of enzymatic activity per liter (U/L). Letters indicate a significant difference between values of each condition using the Tukey (HSD) test at *p* < 0.05. Fermentations were carried out in duplicate.

The ability of each strain to degrade mannoproteins was evaluated in WLM for all *O. oeni* strains. All strains exhibited α-mannosidase activity under wine-like conditions (Figure 3), even without the addition of mannoprotein fractions, which are known to enhance this enzymatic activity [40]. This suggests that α-mannosidase activity is likely constitutive in all tested strains. Notably, *O. oeni* strains LAB6 and LAC20 showed the highest α-mannosidase activity (Figure 3).

**Figure 4:**
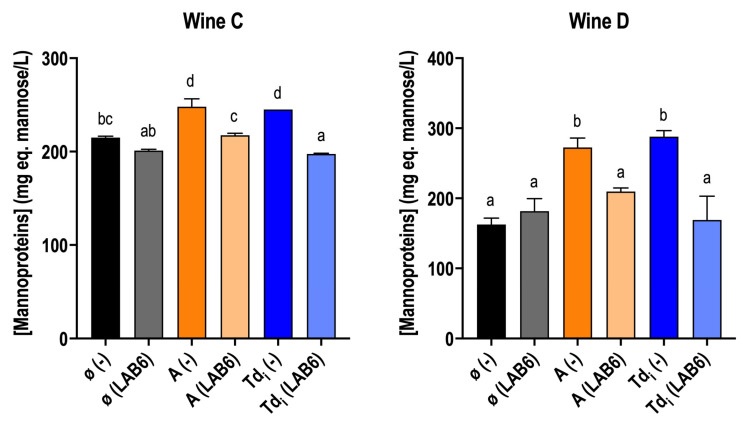
Mannoprotein concentration (mg mannose eq./L) in wine C and D supplemented or not (ø) with *T. delbrueckii* fractions (Tdi and A), inoculated with *O. oeni* (LAB6) or with no inoculation (−). Measurements are from wines after MLF, in case they finished with the addition of the extracts, or after 40 days. (−) wines were taken as a reference wine without inoculation to compare the potential mannoprotein consumption by *O. oeni*. Letters mean that values are significant at *p* < 0.05. Fermentations were carried out in duplicate.

The mannoprotein concentration in these wines, C and D, was determined (Figure 4). The supplementation of fractions Td_i_ and A increased mannoprotein concentration in wines without *O. oeni* inoculation in both wines. Interestingly, a decrease in mannoprotein concentration was observed in those supplemented wines, but not in the control wine. This could be related to the low viability (Table 3) and metabolic activity observed in the MLF performance (Figure 2). Mannoprotein consumption has been reported to be enhanced with higher concentrations [38] under wine conditions, as it was observed in this experiment (Figure 4). In addition, mannoprotein concentration was higher in wine D compared to wine C, as it was the mannoprotein consumption.

**Figure 5:**
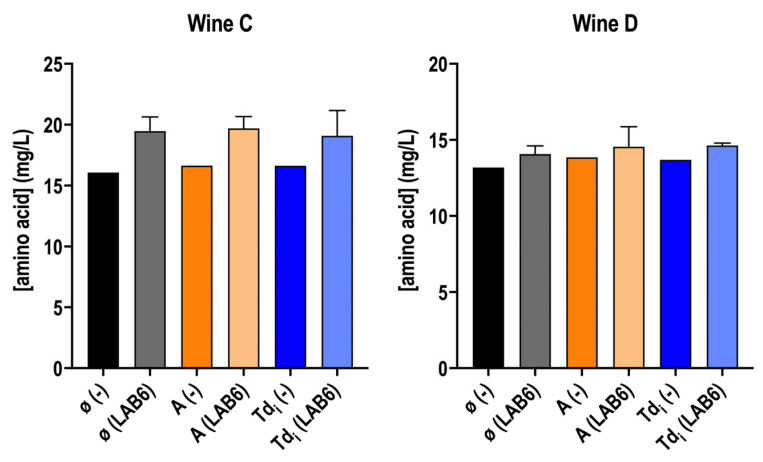
Total free amino acid concentration in wine C and D supplemented or not (ø) with *T. delbrueckii* fractions (Td_i_, and A), inoculated with *O. oeni* (LAB6) or with no inoculation (−). Measurements are from wines after MLF, in case they finished—with the addition of the fractions—or after 40 days. (−) wines were taken as a reference wine without inoculation to compare the potential mannoprotein consumption by *O. oeni*. Fermentations were carried out in duplicate.

Amino acid concentration was also determined. Nitrogen compounds are not abundant in wine after AF. Indeed, there is a complex dynamic since amino acids can be consumed by LAB, while they are again released from peptides or proteins [54,57,58,59]. The free amino acid concentration is usually maintained after MLF, compared to the concentration found before this fermentation [57,58]. In this experiment, the results align with the literature, as no significant changes were observed in these wines regardless of whether *O. oeni* was inoculated or not (Figure 5). Even so, a trend can be observed, mainly in wine C, where wines after MLF showed a slight increase in free amino acids, which could indicate hydrolysis from peptides [44,54] prior to their consumption.

Considering the obtained results, the Chardonnay wine, wine D, was selected for testing the other four *O. oeni* strains with the Td_i_ extract. In this trial, the fraction also showed its potential for reducing MLF duration with all five tested strains (Table 4). Indeed, the control wine did not finish MLF (Table 4). When adding the Td_i_ fraction, all strains could finish the MLF. Similar durations, about 20 days, were obtained with LAA4, LAC20, and LAB9 strains. LAA1 and LAB2013 showed more extended durations. Once again, confirming the potential of non-*Saccharomyces* yeasts for promoting *O. oeni* growth and MLF, not only as a living starter culture but also as an activator extract for MLF [36].

Indeed, the effect on maintaining or even promoting bacterial viability was also observed in this confirmation test. All tested strains showed increased viable population in wines supplemented with the Td_i_ extract after 13 and 20 days of fermentation (Table 4), aligned with the previous observed results of LAB6 in wines C and D (Table 3). The observed increase in viability was up to 1 log in most strains and at most sampling points, except for day 6 of fermentation. This is consistent with previous findings that reported a positive effect associated with prolonged fermentation durations (Table 3). Nevertheless, no significant decrease in viability was observed in relation to the supplementation with the Td_i_ fraction.

Altogether, it was confirmed that the supplementation with *T. delbrueckii* fractions improved MLF performance by reducing the fermentative duration (Figure 1 and Figure 2). This was consistent for different types of wine and for different *O. oeni* strains (Figure 5). From all four extracts, the best results were obtained with the Td_i_ and A extracts (Figure 1). The monitoring of bacterial viability during the first two weeks of fermentation showed that viability was maintained in those supplemented wines (Table 3), which could suggest a survival enhancement for *O. oeni* [46]. In this regard, an increase in mannoprotein consumption was observed in those supplemented wines (Figure 4), which is also related to better adaptation to wine conditions.

The study provides insights into how specific fractions can influence bacterial behavior, offering potential technological applications for promoting MLF in harsh conditions. Future work should address sensory and aroma evaluations, test these fractions at pilot and industrial scale, to evaluate the feasibility and applicability in winemaking.

## 4. Conclusions

In this study, different fractions of *T. delbrueckii* were used to evaluate their potential to improve MLF in wine. The red and white wines selected for the experiment represented harsh conditions for MLF, as no spontaneous MLF occurred in any of the wines. However, supplementation with the four tested fractions—particularly Td_i_ and A—enabled MLF when a commercial *O. oeni* strain was inoculated. Under these conditions, the commercial strain stuck in the wines without Td_i_ fraction supplementation, leaving more than half of the initial L-malic acid unconsumed. This outcome was confirmed in three independent experiments. Notably, the viability of *O. oeni* improved two weeks after inoculation in wines supplemented with the extracts. In addition, mannoprotein consumption was detected in the supplemented wines, suggesting a potential detoxifying role of the extracts and a possible enhancement of *O. oeni* survival mechanisms. These results support the potential application of *T. delbrueckii*-derived fractions, and not only as a living starter culture, mainly Td_i_ and A fractions, as enological tools, which can promote and facilitate MLF under harsh wine conditions, thus saving time and ensuring the completion of the process. Further research will focus on larger-scale trials and the evaluation of sensory, technological, and practical outcomes to better understand the potential of these fractions in winemaking.

## Figures and Tables

**Table 1:** Wines used in this study with some oenological parameters. * PAN—Primary Amino Nitrogen.

Cultivar	Wine A Merlot	Wine B Merlot	Wine C Tempranillo	Wine D Chardonnay
Ethanol (% *v*/*v*)	13.5	14.9	16.2	14.2
pH	3.20	3.30	3.53	3.20
Residual sugars (g/L)	<0.4	<0.4	<0.4	<0.4
L-malic acid (g/L)	2.06	0.99	3.21	2.55
Citric acid (mg/L)	126	217	180	141
Acetic acid (g/L)	0.25	0.52	0.35	0.49
D-lactic acid (g/L)	0.11	0.08	0.22	0.10
L-lactic acid (g/L)	0.10	<0.10	0.10	<0.10
Total SO_2_ (mg/L)	<25	<25	<25	<25
Free SO_2_ (mg/L)	<8	<8	<8	<8
PAN * (mg N/L)	11	86	60	37
NH_4_^+^ (mg/L)	10	96	23	11
Color intensity	11.9	17.8	13.2	0.3

**Table 2:** Alcoholic (AF) and malolactic fermentation (MLF) duration (days) in Tempranillo wines from vintage 2023 produced by the inoculation of *S. cerevisiae* (Sc) or a sequential fermentation of *T. delbrueckii* and *S. cerevisiae* (Td + Sc). Oo: *O. oeni*, Sp: spontaneous MLF without LAB inoculation. Fermentations were carried out in triplicate.

	AF	MLF						
		Oo LAA1	Oo LAB6	Oo LAB9	Oo LAB2013	Oo LAC20	Oo LAA4	Sp
Sc	10	15	15	15	15	15	15	44
Td + Sc	16	8	8	8	8	8	8	8

**Table 3:** Viability of LAB population on MRSmf plates (log CFU/mL) in wine C and D, supplemented or not (ø) with *T. delbrueckii* fractions (Td_i_ and A), inoculated with *O. oeni* (LAB6) at different sampling points (t = 2, 7, and 14 days after inoculation). Fermentations were carried out in duplicate.

	ø	Td_i_	A
Wine C			
t_2_	6.5 ± 0.1	6.6 ± 0.1	6.2 ± 0.2
t_7_	7.0 ± 0.1	7.1 ± 0.1	6.9 ± 0.1
t_14_	6.0 ± 0.3 ^a^	7.0 ± 0.1 ^b^	7.3 ± 0.2 ^b^
Wine D			
t_2_	6.5 ± 0.1	6.4 ± 0.1	6.2 ± 0.1
t_7_	6.4 ± 0.1	6.3 ± 0.1	6.2 ± 0.2
t_14_	6.0 ± 0.3 ^a^	7.2 ± 0.3 ^b^	7.6 ± 0.1 ^b^

Superscripts mean that values are significant at *p* < 0.05 between rows (different treatment, sample sampling point). No letter means no significant difference. Statistics were carried out for each wine independently.

**Table 4:** Malolactic fermentation duration and residual average L-malic concentration after 40 days of fermentation of wine D inoculated with *O. oeni* LAA1, LAB2013, LAA4, LAC20, LAB9, and supplemented or not (ø) with the Td_i_ fraction. Spontaneous MLF is not included since it did not occur. -: uncompleted MLF, n.d.: not detected. Fermentations were carried out in duplicate.

*O. oeni* Strain	Duration (Days)		Residual L-Malic Acid (g/L)		Viability After 6 Days (log CFU/mL)		Viability After 13 Days (log CFU/mL)		Viability After 20 Days (log CFU/mL)	
	ø	Td_i_	ø	Td_i_	ø	Td_i_	ø	Td_i_	ø	Td_i_
LAA1	-	42	1.85	n.d.	6.0 ± 0.1	6.1 ± 0.1	5.6 ± 0.1 ^a^	6.0 ± 0.1 ^b^	5.2 ± 0.1 ^a^	5.9 ± 0.1 ^b^
LAB2013	-	35	1.49	n.d.	6.1 ± 0.1	6.2 ± 0.1	5.4 ± 0.1	5.6 ± 0.1	4.9 ± 0.1 ^a^	5.8 ± 0.1 ^b^
LAA4	-	20	1.25	n.d.	6.2 ± 0.1	6.4 ± 0.1	5.9 ± 0.1 ^a^	7.2 ± 0.1 ^b^	5.8 ± 0.1 ^a^	6.6 ± 0.1 ^b^
LAC20	-	20	1.10	n.d.	6.2 ± 0.1	6.2 ± 0.1	6.1 ± 0.1 ^a^	7.1 ± 0.1 ^b^	5.9 ± 0.1	5.7 ± 0.1
LAB9	-	20	1.35	n.d.	6.2 ± 0.1	6.4 ± 0.1	6.0 ± 0.1 ^a^	7.4 ± 0.1 ^b^	6.4 ± 0.1	6.6 ± 0.1

Superscripts mean that values are significant at *p* < 0.05 between rows (presence or not of Td_i_, sample sampling point, same *O. oeni* strain). No letter means no significant difference. Statistics were carried out for each sampling point and bacterial strain independently.

## Data Availability

The original contributions presented in this study are included in the article. Further inquiries can be directed to the corresponding author.

## Appendix A

**Table A1:** Duration of MLF in wines A, B, C, and D inoculated with *O. oeni* LAB6 and the chemical characterization of the obtained wines after MLF. Ø: no fraction. Td_i_, A, YE, and CW mean inactivated yeast extract (Td_i_), autolysate (A), yeast extract (YE), and cell wall extract (CW).

	Duration (d)	L-Malic Acid (g/L)	pH	L-Lactic Acid (g/L)	D-Lactic Acid (g/L)	Citric Acid (mg/L)	Acetic Acid (g/L)	Glycerol (g/L)	PAN (mg N/L)	NH_4_^+^ (mg N/L)
A-Ø		~0.20 ^b^	3.19 ± 0.01 ^a^	1.13 ± 0.01 ^a^	0.14 ± 0.01 ^ab^	77 ± 1	0.29 ± 0.02 ^a^	7.30 ± 0.57 ^ab^	8 ± 2 ^ab^	22 ± 2 ^b^
A-Td_i_		~0.16 ^a^	3.19 ± 0.01 ^a^	1.12 ± 0.03 ^a^	0.13 ± 0.01 ^a^	77 ± 8	0.28 ± 0.02 ^a^	7.10 ± 0.01 ^a^	8 ± 1 ^a^	26 ± 6 ^b^
A-A	61	n.d.	3.31 ± 0.01 ^b^	1.27 ± 0.05 ^b^	0.15 ± 0.01 ^bc^	56 ± 1	0.40 ± 0.02 ^b^	8.45 ± 0.07 ^ab^	12 ± 2 ^ab^	15 ± 1 ^ab^
A-YE	61	n.d.	3.30 ± 0.01 ^b^	1.18 ± 0.04 ^ab^	0.15 ± 0.01 ^c^	61 ± 20	0.37 ± 0.01 ^b^	8.50 ± 0.28 ^b^	14 ± 1 ^b^	9 ± 1 ^a^
A-CW		~0.24 ^c^	3.18 ± 0.01 ^a^	1.11 ± 0.01 ^a^	0.13 ± 0.01 ^a^	79 ± 1	0.28 ± 0.01 ^a^	7.40 ± 0.42 ^ab^	14 ± 1 ^b^	9 ± 2 ^a^
B-Ø		~0.42	3.32 ± 0.01 ^a^	0.36 ± 0.01 ^a^	0.12 ± 0.04	192 ± 11	0.46 ± 0.01	8.85 ± 0.07 ^a^	65 ± 1 ^a^	57 ± 3 ^a^
B-Td_i_	21	n.d.	3.42 ± 0.04 ^b^	0.58 ± 0.03 ^c^	0.12 ± 0.01	209 ± 1	0.52 ± 0.01	9.40 ± 0.14 ^ab^	70 ± 1 ^a^	68 ± 2 ^b^
B-A	24	n.d.	3.44 ± 0.02 ^b^	0.54 ± 0.02 ^bc^	0.11 ± 0.01	201 ± 5	0.51 ± 0.04	9.45 ± 0.07 ^ab^	72 ± 1 ^ab^	61 ± 1 ^ab^
B-YE	24	n.d.	3.45 ± 0.01 ^b^	0.50 ± 0.02 ^b^	0.11 ± 0.01	199 ± 11	0.50 ± 0.01	9.45 ± 0.07 ^ab^	86 ± 5 ^c^	63 ± 10 ^ab^
B-CW	41	n.d.	3.45 ± 0.02 ^b^	0.54 ± 0.01 ^bc^	0.11 ± 0.01	199 ± 23	0.51 ± 0.03	9.70 ± 0.28 ^b^	83 ± 4 ^bc^	68 ± 2 ^ab^
C-Ø		~2.00 ^b^	3.70 ± 0.01	0.77 ± 0.01 ^a^	0.25 ± 0.02 ^a^	280 ± 11	0.34 ± 0.01 ^a^	8.90 ± 0.01	43 ± 1 ^a^	22 ± 5
C-Td_i_	45	n.d.	3.82 ± 0.11	2.07 ± 0.11 ^b^	0.38 ± 0.02 ^abc^	210 ± 6	0.42 ± 0.01 ^b^	9.35 ± 0.21	49 ± 1 ^ab^	44 ± 4
C-A	24	n.d.	3.80 ± 0.14	2.14 ± 0.07 ^b^	0.42 ± 0.03 ^c^	251 ± 3	0.41 ± 0.01 ^b^	9.50 ± 0.01	52 ± 3 ^ab^	34 ± 8
C-YE		~1.81 ^a^	3.72 ± 0.01	0.91 ± 0.01 ^a^	0.26 ± 0.05 ^ab^	283 ± 39	0.35 ± 0.01 ^a^	8.80 ± 0.14	57 ± 3 ^b^	25 ± 1
C-CW	27	n.d.	3.84 ± 0.08	2.05 ± 0.15 ^b^	0.42 ± 0.06 ^bc^	242 ± 8	0.41 ± 0.02 ^b^	9.50 ± 0.57	58 ± 4 ^b^	27 ± 3
D-Ø		~1.70 ^b^	3.29 ± 0.01 ^a^	0.58 ± 0.05 ^a^	0.11 ± 0.01 ^a^	182 ± 14 ^c^	0.49 ± 0.01	9.55 ± 0.07	35 ± 1	n.d.
D-Td_i_	17	n.d.	3.46 ± 0.01 ^b^	1.63 ± 0.10 ^c^	0.13 ± 0.01 ^ab^	149 ± 11 ^abc^	0.56 ± 0.01	9.35 ± 0.21	31 ± 6	7 ± 3
D-A	17	n.d.	3.47 ± 0.01 ^b^	1.67 ± 0.16 ^c^	0.13 ± 0.01 ^ab^	145 ± 4 ^ab^	0.57 ± 0.05	9.70 ± 0.14	36 ± 8	n.d.
D-YE		~1.19 ^a^	3.30 ± 0.01 ^a^	0.96 ± 0.04 ^b^	0.12 ± 0.01 ^a^	178 ± 1 ^bc^	0.55 ± 0.04	9.65 ± 0.35	41 ± 1	n.d.
D-CW	21	n.d.	3.48 ± 0.01 ^b^	1.67 ± 0.01 ^c^	0.15 ± 0.01 ^b^	131 ± 5 ^a^	0.58 ± 0.01	9.65 ± 0.35	37 ± 1	8 ± 1

Superscripts mean that values are significant at *p* < 0.05. No letter means no significant difference. Statistics were conducted for each wine independently.
